# pyam: Analysis and visualisation of integrated assessment and macro-energy scenarios

**DOI:** 10.12688/openreseurope.13633.2

**Published:** 2021-09-01

**Authors:** Daniel Huppmann, Matthew J. Gidden, Zebedee Nicholls, Jonas Hörsch, Robin Lamboll, Paul N. Kishimoto, Thorsten Burandt, Oliver Fricko, Edward Byers, Jarmo Kikstra, Maarten Brinkerink, Maik Budzinski, Florian Maczek, Sebastian Zwickl-Bernhard, Lara Welder, Erik Francisco Álvarez Quispe, Christopher J. Smith

**Affiliations:** 1 Energy, Climate and Environment Program (ECE), International Institute for Applied Systems Analysis (IIASA), Laxenburg, Austria; 2 Climate Analytics, Berlin, Germany; 3 Climate & Energy College, University of Melbourne, Melbourne, Australia; 4 School of Geography, Earth and Atmospheric Sciences, University of Melbourne, Melbourne, Australia; 5 The Grantham Institute for Climate Change and the Environment, Imperial College London, London, UK; 6 Workgroup for Infrastructure Policy, Technische Universität Berlin, Berlin, Germany; 7 Centre for Environmental Policy, Imperial College London, London, UK; 8 MaREI Centre, Environmental Research Institute, University College Cork, Cork, Ireland; 9 Department of Energy and Process Engineering, Norwegian University of Science and Technology, Trondheim, Norway; 10 Graz University of Technology, Graz, Austria; 11 Energy Economics Group (EEG), Technische Universität Wien, Vienna, Austria; 12 Institute of Technological Research (IIT), Comillas Pontifical University, Madrid, Spain; 13 School of Earth and Environment, University of Leeds, Leeds, UK

**Keywords:** integrated assessment, energy systems, macro-energy, modelling, scenario analysis, data visualisation, Python package

## Abstract

The open-source Python package pyam provides a suite of features and methods for the analysis, validation and visualization of reference data and scenario results generated by integrated assessment models, macro-energy tools and other frameworks in the domain of energy transition, climate change mitigation and sustainable development. It bridges the gap between scenario processing and visualisation solutions that are "hard-wired" to specific modelling frameworks and generic data analysis or plotting packages.

The package aims to facilitate reproducibility and reliability of scenario processing, validation and analysis by providing well-tested and documented methods for working with timeseries data in the context of climate policy and energy systems. It supports various data formats, including sub-annual resolution using continuous time representation and "representative timeslices".

The pyam package can be useful for modelers generating scenario results using their own tools as well as researchers and analysts working with existing scenario ensembles such as those supporting the IPCC reports or produced in research projects. It is structured in a way that it can be applied irrespective of a user's domain expertise or level of Python knowledge, supporting experts as well as novice users.

The code base is implemented following best practices of collaborative scientific-software development. This manuscript describes the design principles of the package and the types of data which can be handled. The usefulness of pyam is illustrated by highlighting several recent applications.

## Introduction

### Towards open-source tools in energy & climate modelling

Over the past years, the scientific communities for energy systems modelling and integrated assessment of climate change mitigation pathways have made significant strides to “#freethemodels” ^1, 2^. This includes steps to release input data, assumptions, algebraic formulation, and processing tools for scenario results under open-source licenses, in order to facilitate transparency and reproducibility of scientific analysis. These efforts are part of a larger push towards open science and FAIR data management principles (Findable, Accessible, Interoperable, Reusable ^3^) supported by stakeholders, funding agencies and researchers themselves, for example the openmod initiative. 

Alas, the efforts to move to open-source and collaborative (scientific) software development practices in energy systems modelling, macro-energy research and integrated assessment have, so far, mostly focused on modelling frameworks and input data. The processing of scenario results using a common set of tools and methods has received much less attention. In many cases, users are either confined to tools for processing of results that are highly customized to a specific modelling framework, or they have to develop their own methods and scripts using general-purposes packages. In a Python environment, for example, users often write their own workflows and analysis tools from scratch using pandas, numpy, matplotlib ^4^ and seaborn ^5^.

The vision of **pyam** is to bridge that gap: to provide a suite of features and methods that are applicable for scenario processing, analysis and visualization irrespective of the modelling framework. At the same time, the package should be sufficiently specific for energy systems modelling as well as integrated assessment of climate change and sustainable development to allow sensible defaults and remove as much clutter as possible from scenario processing workflows or analysis scripts. Using a standardized, well-structured toolbox rather than own custom methods can also reduce the scope for errors and improve the reliability and readability of scenario processing code.

### An overview of existing packages and tools

Several open-source packages and tools exist in between the general-purpose packages for data analysis and plotting, on the one hand, and dedicated data processing solutions specifically built around a specific modelling framework, on the other hand.

**Figure 1.**
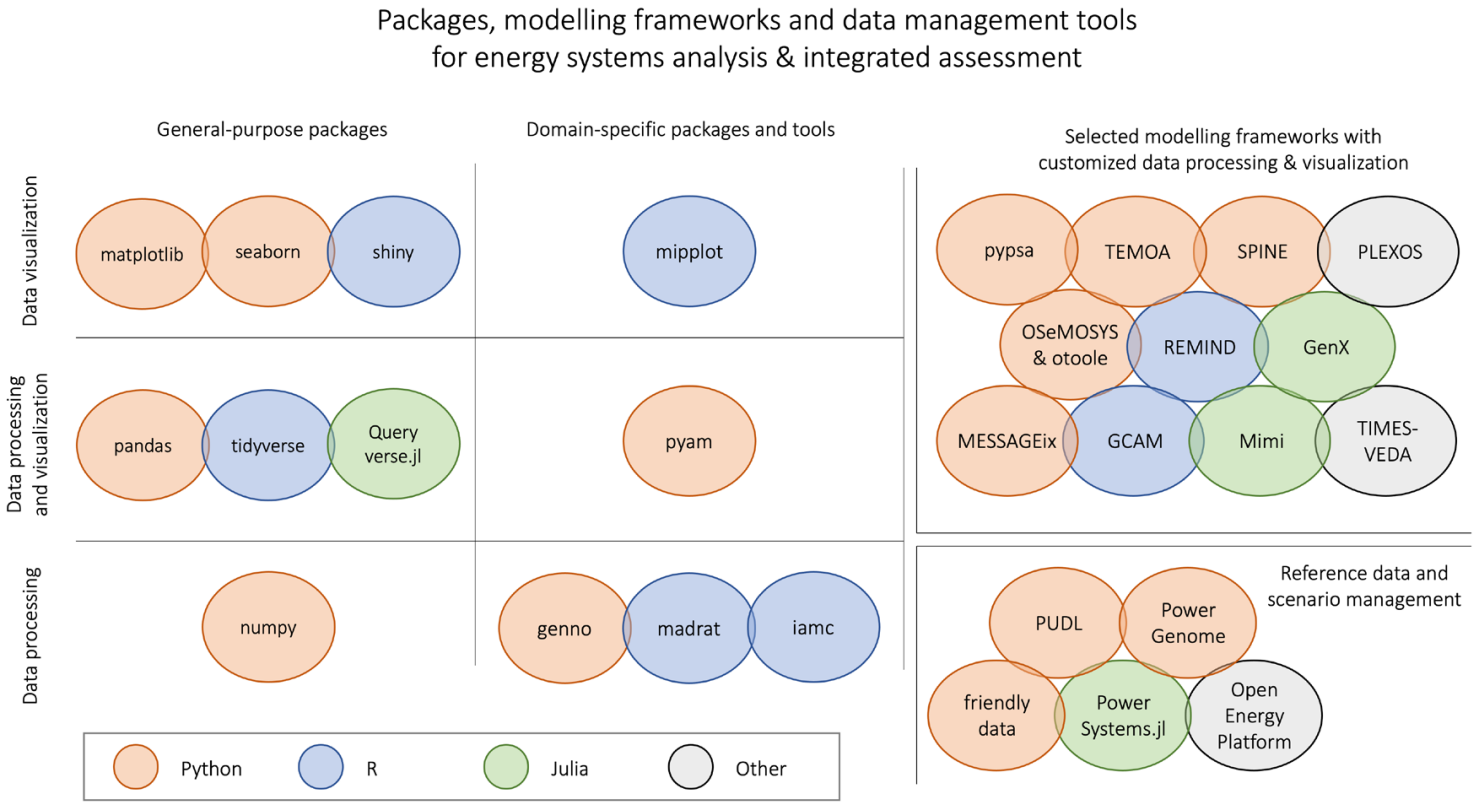
Overview of packages & tools for energy system & integrated assessment modelling. (see the Appendix for a full list of references and links cited in this figure).

The packages on the left-hand side of Figure 1 are powerful, general-purpose, domain-agnostic solutions for data science. In contrast, in the top-right corner is a selection of several widely used modelling frameworks that come with dedicated analysis and visualization features "hard-wired" to their implementation.

In the middle of the figure are several packages and tools that are not customized to any particular modelling framework, but are geared for broader use in the domain of energy systems and integrated assessment modelling. These packages are compatible with a variety of data formats commonly used by the respective research communities.

The R package madrat provides a framework for improving reproducibility and transparency in data processing. It enables the definition and execution of workflows that are frequently nused in this research domain ^6^. In comparison, the R package iamc is a collection of functions for data analysis and diagnostics of scenario results in the IAMC format (see the following section on data models for more information). The Python package genno supports describing and executing complex calculations on labelled, multi-dimensional data; it was developed as a generalization of data processing in the context of integrated assessment and transport modelling.

In contrast, the R package mipplot is a solution for visualization of scenario results related to climate mitigation ^7^. It is also based on the IAMC format.

The pyam package, similar to the pandas package in the general-purpose "column" of the figure, provides features and methods both for data processing as well as for visualization and plotting. It was developed specifically for supporting workflows and conducting analysis for input data for and results from energy system models like those shown in the top-right corner of the figure.

As one additional group of relevant packages for the energy systems modelling domain, the figure shows several tools for reference data compilation (model input) and storage of scenario results (model output):

 The Public Utility Data Liberation ( PUDL) project takes publicly available information and makes it usable by cleaning, standardizing, and cross-linking utility data from different sources in a single database. In a similar effort, PowerGenome compiles different data sources into a single database.

The friendly_data_ package implements an adaptation to make the frictionless datapackage standard more easily usable in the energy systems domain. The PowerSystems.jl package provides a rigorous data model to enable power systems analysis and modelling across several input formats. The Open Energy Platform aims to ensure quality, transparency and reproducibility in energy system research. It is a collaborative community effort to develop various tools and information that help working with energy-related data.

These tools are valuable to facilitate the use of consistent data when calibrating or evaluating models, and they simplify the process to share and compare results across modelling frameworks. Alas, these tools still suffer from fragmentation and incompatible data formats. To integrate them with either a general-purpose data science package or a specific modelling framework requires substantial effort.

### A Python package for scenario analysis & visualization

We believe that pyam can serve a useful "bridge" between different modelling frameworks, or between models and various data management solutions. Due to its wide scope encompassing various aspects of data science and visualization options, it can be a valuable addition to the to the suite of tools used by the energy systems and integrated assessment modelling communities.

The pyam package grew out of complementary efforts in the Horizon 2020 project CRESCENDO and the analysis of integrated-assessment scenarios supporting the IPCC’s *Special Report on Global Warming of 1.5°C*. An earlier manuscript describes describes its features and capabilities at that time ^8^. After more than two years of further development, we believe that the package has now reached a reasonable level of maturity to be useful to a wider audience - in scientific-software jargon, it is ready for **release 1.0**.

The aim of the package is not to provide complex new methodologies or sophisticated plotting features. Instead, the vision is to provide a toolbox for many small operations and processing steps that a researcher or analyst frequently needs when working with numerical scenarios of climate change mitigation and the energy system transition: aggregation & downscaling, unit conversion, validation, and a simple plotting library to quickly get an intuition of the scenario data.

The package can be used for results generated from any model in the listed domains above or related reference sources, if the data does have some sectoral, spatial and temporal dimension. While we use the term "timeseries" throughout this manuscript, pyam can handle data that has only one level of regional and temporal resolution, e.g., global CO2 emissions in one specific year.

By following the design of pandas and other mature, well-established packages, it can appeal to a broad range of user groups:

• Modelers generating scenario results using their own tools and frameworks, as well as researchers and analysts working with existing scenario ensembles such as those supporting the IPCC reports or produced in research projects.

• Users that want to add a particular step to an existing scenario processing workflow as well as modelers that are starting scenario analysis from scratch.

• Python experts as well as novice users of this programming language.

This manuscript describes the design principles of the package and the types of data that can be handled. We present a number of features and recent applications to illustrate the usefulness of pyam, and we point to the tutorials that can help potential users to decide whether the pyam package may be suitable for them. In the last section, we identify several forthcoming uses cases and planned developments.

## Data models and formats used by the energy & climate modelling communities

When researchers in the domain of energy modelling and climate science hear the term “model", they usually think of numerical tools to compute results from given inputs. This section is about a different type of model.

A “data model” is an abstract description of the structure of information. It can refer to timeseries data, static characteristics of technologies or resources, or any other numerical information. In its essence, a table with clear rules on the kind of values in each column is already a data model.

Numerous concepts are in use in the domain of energy systems modelling and climate science to store reference data, facilitate exchange of data between models, or make results available to other users.. This section describes several commonly used concepts and related formats in the integrated-assessment community as well as energy systems, macro-energy and climate modelling.

### The IAMC format

A decade ago, the *Integrated Assessment Modeling Consortium* ( IAMC) established a simple tabular template to exchange yearly timeseries data related to energy systems modelling, land-use (change), demand sectors, and economic indicators in the context of climate change mitigation scenarios. Previous high-level use cases include reports by the *Intergovernmental Panel on Climate Change* (IPCC, ^9^) and model comparison exercises within the *Energy Modeling Forum* ( EMF) hosted by Stanford University.

The tabular format consists of the columns *model*, *scenario*, *region*, *variable* and *unit* as well as one column per year. The IAMC also introduced conventions on the structure of the identifiers. Most importantly, the *variable* column describes the type of information represented in the specific timeseries. It implements a “semi-hierarchical” structure using the | character ( *pipe*, not l or i) to indicate the *depth*. Variable names (should) follow a structure like *Category|Subcategory|Specification*.

Semi-hierarchical means that a hierarchy can be imposed, e.g., a user can specify that the sum of *Emissions|CO2|Energy* and *Emissions|CO2|Other* must be equal to *Emissions|CO2* (if there are no other *Emissions|CO2|...* variables). However, this is not always mandatory: for example, the sum of *Primary Energy|Coal*, *Primary Energy|Gas* and *Primary Energy|Fossil* should not be equal to *Primary Energy* because this would double-count fossil fuels.

### The openENTRANCE extensions of the IAMC format

The Horizon 2020 project openENTRANCE adapted the IAMC data template and extended it in two directions to make the format better suited for energy systems modelling. Specifically, this requires a more detailed representation of subannual data and a better solution to represent trade flows and similar inter-regional quantities, i.e., timeseries for data that is defined on the connection between two regions.

To this end, the openENTRANCE project introduced a *subannual* column to the IAMC format to describe data at a subannual resolution: the entries of that column can be identifiers like “Summer” or “January”, or timestamps stripped of the “year” component, e.g., “01-01 06:00:00+01:00” for January 1st, 6 am in the Central European time zone (the year information remains in the columns of the tabular data.)

The second extension concerns *directional* information, e.g., trade flows or energy transmission from one region to a neighbouring country. A > sign in the region column can be used to indicate the source and destination of the timeseries in that row, e.g., *Region A>Region B*.

To facilitate the adoption and usage of these conventions, the openENTRANCE consortium developed an installable Python package. This includes the lists of variables, regions and units used in the project to exchange data between models, and it provides utility functions to validate that a dataset conforms to the common definitions.

### Formats for power sector modelling

One relatively early and widely used set of open-source tools for electric power system simulation and optimization is MATPOWER ^10^, implemented in MATLAB. Its data model, the “MATPOWER case format”, holds technical and economical parameters of a power system made of buses, branches, generators and storage units for one particular snapshot in time.

Subsequent open-source implementations of power system modelling frameworks and tools like the Python-based PyPSA ^11^ or pandapower ^12^ or the Julia-based PowerSystems.jl package each prefer their own NetCDF, CSV or JSON-based formats to store time-series data, but most of them include importers for the MATPOWER case format to easily use the suite of test networks available in that format. The industry standards CIM (Common Information Format) or PSS/E’s “RAW” formats have found less adoption in the scientific community ^13^.

### Data formats and standards in the climate science community

Within the climate science community, a widespread and well-known data model is that of the Coupled Model Intercomparison Project (CMIP, ^14, 15^). The data model is designed to handle the enormous CMIP data volumes (approximately 18PB, ^16^) generated with participation from dozens of modelling teams and to ensure consistency across many sub-disciplines of earth sciences and experimental setups. It has traditionally revolved around the netCDF format and the CF metadata convention, a self-describing binary format designed for array-oriented scientific data ^17^ commonly used for earth sciences data. The data is organised according to a regularised data reference syntax ^16^, which splits the data into smaller pieces that can be reasonably handled by climate science: the dimensions include the experiment performed, the model that performed the experiment, the experiment realisation (not all realisations are the same because the models include chaotic dynamics) and the version of the output.

One major challenge is often simply accessing the data, for which substantial computation is normally required. Increasingly, scientists are moving their analysis workflows to high-performance cloud computing platforms. This allows to host up-to-date data and supports containerized environments such as Pangeo and Google Earth Engine. 

A number of tools have been developed over the years to work specifically with climate data: NCL and CDO ^18^ are the most popular command line options. More recently, the popularity of Python and its ease of working with large multi-dimensional arrays in xarray ^19^ and Dask has led to a growing geosciences ecosystem in that programming language. This includes climate-specific packages such as Iris ^20^ and the ESMValTool ^21^, which builds on Iris in an effort to create reproducible climate-data analysis workflows whilst also allowing researchers to build on each other’s data processing efforts, particularly related to parallelisation and lazy data handling. It should be noted that the ESMValTool supports programming languages other than Python, with the aim of being as open as possible.

### Bridging the gap between integrated assessment and climate science

Beyond the CMIP archive, there are a myriad of other data formats and conventions within the climate literature. Of these, the most relevant to the integrated-assessment community is scmdata ^22^. Being built with the IAMC data format (see above) in mind, scmdata uses completely interoperable conventions and an identical data format, most notably in the structure of the *variable* column. The close link between scmdata and pyam facilitates the integration between integrated-assessment models and reduced complexity climate models. This linkage is already widely used in projects involving IAMC member institutions and the assessment by Working Group 3 of the IPCC. To extract data from the CMIP archive into the scmdata format, the package netCDF-SCM was developed ^23^.

The pyam package was initiated based on the IAMC format and the work done to foster the link between the integrated-assessment community and the climate sciences. The following section describes the design principles of the package and the generalized data model for which it can be applied.

## The pyam package

### Design principles, implementation and user groups

The vision for the pyam package is to provide a toolbox for many small operations and processing steps that a researcher or analyst frequently needs when working with numerical scenarios of climate change mitigation and the energy system transition. The central paradigm for implementing this aim is to leverage the structure of the data model (see the following section) for common operations such as unit conversion or aggregation along sectoral, regional and temporal dimensions.

We see this package as serving several distinct groups: *experienced Python* users, whose natural tendency would be to reimplement any data processing step directly in a general-purpose data analysis package like pandas or numpy; and users with *domain expertise but limited Python knowledge*, who appreciate a package enabling a wide range of data processing actions and providing simple commands to perform common tasks. 

The package should be equally suitable for modellers generating scenario results with their own modelling frameworks, as well as researchers or analysts working with scenario ensemble data compiled by other research groups or institutions. Also, it is important to remain aware that users will always have requirements that cannot be realistically met by any single package. Therefore, the implementation should support a modular approach and efficient integration of the pyam package with other tools for data processing and analysis.

To reconcile these competing interests, we decided to follow the design of the pandas package as closely as possible. First, the pyam package implements functions that mimic pandas (e.g., rename(), filter()), and it uses similar keyword arguments where possible (e.g., inplace). This makes pyam intuitive for experienced users, and it sets Python novices on a good path when learning more advanced packages later. Second, the pyam implementation is not a monolith; it is structured so that a user can easily use the pyam functionality for parts of a processing workflow, then pull out the internal data objects for more advanced manipulation with pandas or numpy, and then continue with pyam functions.

To further accommodate the alternative user groups, we implemented several tools for community engagement: experienced users will find it most convenient to interact via the GitHub repository; for users with limited experience in collaborative (scientific) software development, an email list hosted by groups.io and a Slack channel provide a less daunting avenue to ask questions or suggest new features.

The pyam package follows widely accepted principles of best practice in scientific software development ^24^. It is released under the open-source APACHE 2.0 license, and the package is available via both pypi.org and conda-forge. Comprehensive documentation is rendered on ReadTheDocs.org. 

The code base is hosted on GitHub to take advantage of its tools for version control and collaboration, and the code follows the Black style, which is the state-of-the-art utility for linting and formatting in Python. It includes an extensive test suite with coverage >90%, executed via GitHub Actions on several operating systems and Python versions for every pull request. Tests are also executed on a regular basis (weekly or nightly) to guard against issues caused by dependency updates.

### The pyam data model

There is an inherent ambiguity about the use of term “scenario” in the community: it can refer to a “scenario protocol”, a set of assumptions or constraints that define a storyline or pathway; it can also refer to the implementation of a scenario protocol in a specific numerical modelling framework, which is then called a “scenario run”.

For example, in the Horizon 2020 CD-LINKS project (started in 2015), researchers agreed on a protocol for a "NPi2020-1000" scenario, assuming that each region or country maintains implemented policies until 2020 and then follows a pathway limiting cumulative global greenhouse gas emissions until the end of the century to 1000 Gt CO2-equivalent. This protocol was then implemented in six numerical modelling frameworks (MESSAGEix-GLOBIOM, REMIND-MAgPIE, etc.). Therefore, in the `IAMC 1.5°C scenario explorer`_, there are six alternative implementations (i.e., runs) of the "NPi2020-1000" scenario protocol.

### The IamDataFrame class

The pyam data model follows the structure of the IAMC format introduced in the previous section, but it generalises its design to support a broader range of use cases. An **IamDataFrame** is a structured collection of numerical implementations of scenarios (i.e., scenario runs).

Each scenario is identified by an index; the standard index dimensions are ‘model’ and 'scenario', where the scenario identifier is understood as a scenario protocol (as explained above) or another descriptive name. The model identifier usually refers to one of the following types:

- an integrated assessment, macro-energy or energy systems model
- a (simple) climate model
- a reference data source, e.g., IEA Statistics for historical data
- a descriptor of the way multiple models were aggregated to make this timeseries (e.g. multi-model mean)

***Timeseries data*.** Each timeseries data point is identified by the index dimensions of the IamDataFrame, the *coordinate* columns ‘region’, ‘variable’, ‘unit’, and a temporal coordinate. The time domain can be yearly data (‘year’) or a continuous date-time format (‘time’) to work with sub-annual data, e.g., scenarios with hourly resolution. It is also possible to add *extra-columns* when more fine-grained indexing is required. This feature can be used to describe “representative timeslices” (e.g., *summer-day*, *peak-hour*), meaning a non-consecutive temporal disaggregation domain.

The internal handling of timeseries data is implemented in *long format*, i.e., a pair of columns for the *value* and the time domain in the data table. Alas, it is often more convenient to display and store timeseries data in *wide format*, where the temporal dimension is displayed as columns. The method timeseries() returns the data in this format as a pandas.DataFrame, and writing to file (see the section “Supported file formats”) applies it, too.

An illustrative example of a ‘data’ table in a standard IAMC wide format is shown in Table 1. It is taken from the IAMC 1.5°C Scenario Explorer ^25^ showing a timeseries data row of a scenario from the CD-LINKS project.

**Table 1.** Illustrative example of a timeseries ‘data’ table in *wide format*.

model	scenario	region	variable	unit	2005	2010	2015	. . .
MESSAGE	CD-LINKS 400	World	Primary Energy	EJ/y	462.5	500.7	. . .	. . .
. . .	. . .	. . .	. . .	. . .	. . .	. . .	. . .	. . .

***Quantitative or qualitative meta indicators*.** Each scenario (i.e., scenario run) can have any number of quantitative or qualitative indicators. The corresponding ‘meta’ table to the example above is shown in Table 2.

**Table 2.** Illustrative example of a ‘meta’ table for quantitative or qualitative scenario indicators.

model	scenario	category	year of peak warming	cumulative CO2	. . .
MESSAGE	CD-LINKS 400	1.5C high overshoot	2051	-17.73	. . .
. . .	. . .	. . .	. . .	. . .	. . .

### Operation and features

The features of the pyam package can by broadly categorized into three groups: scenario processing, validation, and visualization. But before discussing these features, we briefly illustrate how to start working with the package.

***Getting started*.** The pyam package can be used with any kind of scenario results or reference data that has a sectoral, temporal and regional dimension. Even if the dimension only has a unique value (e.g., a global model without regional disaggregation), it often makes sense to specify this information explicitly - in pyam, this would be done by setting the region dimension to "World". This will simplify expanding the level of details later on.

An IamDataFrame can be initialized directly from a pandas DataFrame or an xlsx/csv file. The data must be given in a structure compatible with the pyam data model, but the package will accept numerous implementations and cast it to a valid format. For example, it works with data in wide or long format (see the previous section), and it will takes columns headers that are capitalized ("Model") or not ("model"). It is also possible to pass missing timeseries data columns as keyword arguments, e.g., region="World". The tutorial on data table formats illustrates the various table structures that can be used to initialize an IamDataFrame. There is also a tutorial to read results from a GAMS gdx file for further processing.

In addition to xslx and csv file types, the pyam package also supports reading from and writing to the frictionless datapackage format.

***Scenario processing*.** The most important element of integrated assessment and energy systems modelling apart from the algebraic formulation is the preparation of input data and assumptions as well as the processing of numerical results to a state in which they can be conveniently analysed. The pyam package provides a suite of methods that can facilitate these tasks. Two of them are presented here as illustration of the general implementation strategy.

Input data and modelling results frequently have to be aggregated or downscaled along sectoral, spatial or temporal dimensions. The pyam package provides multiple functions to that effect offering a variety of methods including sum, mean, min and max. In addition, a weighted-average feature can use proxy-variables available at the target resolution directly from the timeseries data, or a weights-dataframe which can be passed as a keyword argument. This enables a user to compute weighted averages with minimal effort, for example using population at a national level as a proxy when downscaling regional energy consumption.


df.downscale_region("Final Energy", proxy="Population")


Alternatively, a user can use a more sophisticated methodology for calculating weights and use pyam only to apply them to the timeseries data using a keyword argument. All of these features call the respective pandas functions on the pyam-internal data object to benefit from the performance and versatility of that package.

For the second illustrative example for data processing, the pyam package provides a method convert_unit(), which uses the iam-units package as a dependency to facilitate intuitive operations. The iam-units package is in turn built on the pint package, a powerful and versatile solution for defining units and performing arithmetic operations on them. pint can natively handle all SI definitions and many other widely used units, and iam-units adds definitions frequently encountered in energy systems, integrated-assessment and climate modelling.

One example of added functionality by the iam-units package is the conversion of greenhouse gas emissions to their CO _2_-equivalent by any of several IPCC Global Warming Potential (GWP) metrics.


df.convert_unit("Mt CH4/yr", to="Gt CO2e/yr", context="AR5GWP100")


Using this package as a dependency in pyam rather than implementing a parallel solution follows the best-practice software design principle of “separation of concerns” and helps to keep the code base as succinct as possible.

***Validation*.** An important part of scenario analysis is the validation of data for completeness and correctness, in particular ensuring that results are close to given reference data or that the sectoral and spatial aggregations are internally consistent. The functions implemented for this purpose are require_variable(), validate(), and several methods with the pattern check_*().

Per default, all validation functions report which scenarios or which data points do not satisfy the respective validation criteria. However, each method also has an option to exclude_on_fail, which marks all scenarios failing the validation as exclude=True in the ‘meta’ table (see the ‘Data Model’ section above). This feature can be particularly helpful when a user wants to perform a number of validation steps and then remove or filter all scenarios violating any of the criteria as part of a scripted workflow.

***Visualization*.** Following the structure of pandas and matplotlib, the pyam package provides direct integration between data manipulation and visualization features. It implements a range of plotting features using matplotlib and seaborn such that users can quickly gain a graphical intuition of the data.

Where possible, the package sets reasonable defaults to streamline the workflow. For example, the simplest possible function call is df.plot() (without any arguments), which draws a line plot using the time domain as the x-axis - this is arguable the most common use case for scenario data.

**Figure 2.**
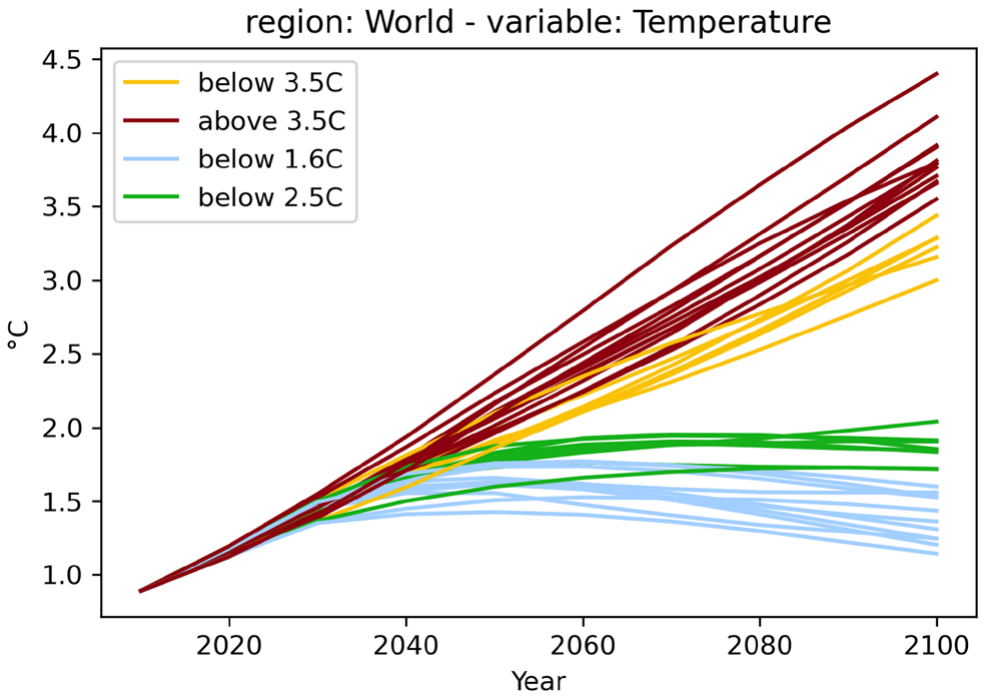
A simple plot from the first-steps tutorial. The plot is created from the code snippet below, the assignment of the *warming-category* and the associated colors is shown in the tutorial notebook.


The plotting library supports all common plot types including (stacked) line, bar and pie charts, boxplots, scatter plots and sankey diagrams. It also supports specifying styles (colors, markers, etc.) grouped by data coordinates or meta indicator, which can then be used directly as arguments in the plotting methods. Figure 2 from the first-steps tutorial illustrates this feature, where warming categories and respective colors have been defined as part of the script.

df.filter(variable="Temperature").plot(color="warming-category")


The pyam package has implementations of several plot types, with a behavior and function signatures following the underlying pandas, matplotlib or seaborn methods. The comprehensive documentation of the plotting functions can be found in the gallery of the documentation.

Last, but not least: by being based on the standard Python plotting libraries matplotlib and seaborn, the pyam plotting functions can be used directly in any more elaborate figure drawn with these packages. This is illustrated in the following code block.


import pyam
import matplotlib.pyplot as plt

df = pyam.IamDataFrame(..)

fig, ax = plt.subplots()
df.plot(ax=ax) # using pyam features to plot data
.. # any other matplotlib features to enhance the figure

fig.show()


### Integration with data resources

To facilitate using external data resources as input data or for validation and plotting of scenario results, pyam supports reading data directly from several databases:

- Any **IIASA Scenario Explorer** instance via the native pyam.iiasa module - see the related tutorial for details. Visit https://data.ece.iiasa.ac.at for a list of project databases hosted by IIASA.
- The **World Bank Development Indicator** database via the pandas-datareader package.
- The **UNFCCC Data Inventory** via the unfccc-di-api package ^26^.
  Refer to the documentation of all functions to query data resources.

## Use cases and applications

### The IPCC Special Report on 1.5°C

**Figure 3.**
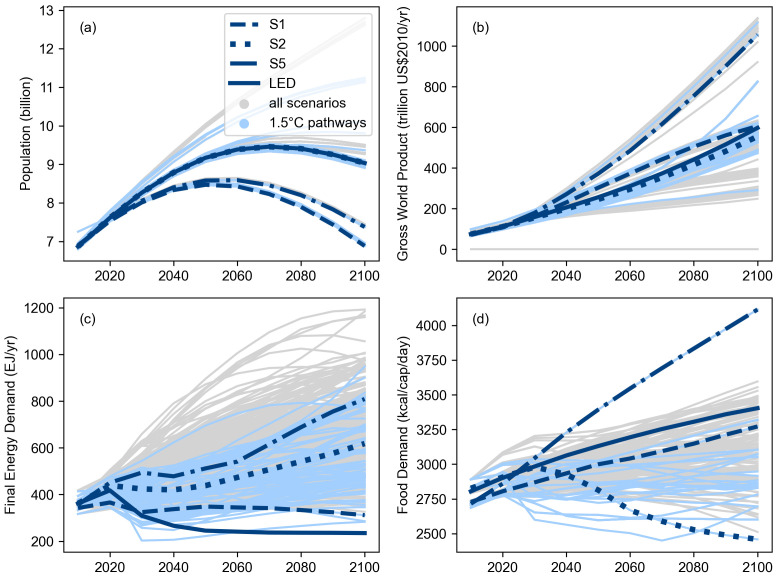
Range of assumptions about socio-economic drivers and projections for energy and food demand as shown in the IPCC SR15 (Figure 2.4, link). The figure was generated using pyam plotting features and the source code was released under an open-source license ( source). The figure is reproduced per the IPCC’s Copyright policy.

The first high-level use of the pyam package was in the assessment of quantitative, model-based pathways in the IPCC’s *Special Report on Global Warming of 1.5°C* (SR15) ^9^. Many of the figures, tables and headline statements in the SR15 were implemented as Jupyter notebooks using an early version of pyam; as illustration, Figure 3 shows a plot from the SR15 created with pyam methods. The notebooks were released under an open-source license to increase transparency and reproducibility of the report ^27^.

### The openENTRANCE nomenclature

The Horizon 2020 project openENTRANCE develops a suite of open-source models to analyse implications and economic costs associated with different energy pathways that Europe could take towards its climate goals. To facilitate the linkage of these models and analyse integrated scenarios, a common data format and an agreed set of naming conventions and definitions for regions, variables and units is required.

This “nomenclature” is implemented collaboratively on GitHub ( repository link) under the open-source Apache 2.0 License. The repository also contains several Python utility functions to validate the consistency of a scenario with the nomenclature. These utility functions are built on the pyam package and its versatility to parse various file formats and data templates. Moreover, a balance between (human) readability and (machine) processability was an important consideration when developing the nomenclature. The common definitions and related validation features will prove useful beyond the project’s scope and can be a cornerstone for future energy model integration.

### Model results processing

Several open-source modelling frameworks started to use the pyam package as part of their processing of model results. Three examples are listed here:

- The GENeSYS-MOD model ^28^ is a variation of the widely used OSeMOSYS framework for capacity planning and energy systems optimization. As part of the model linkage in the openENTRANCE project, the authors implemented a processing workflow using pyam to convert model results to the common data format used in the project. The workflow for this model is available via a central repository storing all model linkage scripts and mappings developed in the openENTRANCE project.
- The GUSTO model ^29^ for the representation and analysis of regional energy communities is based on the urbs model. The processing of model results is currently being reimplemented to use pyam.
- The TEMOA model ^30^ for energy systems analysis includes a prototype implementation to export results to a pyam-compatible Excel file as an alternative to its native data format.

### Model linkage framework

Ref 31 implements a soft-linking framework that supports a workflow between a global integrated-assessment model (IAM) and a detailed power system model. The scenario results from the full-economy model can be fed into the power system model to assess the scenario with enhanced spatial, technological, and temporal resolution. Results from the power system model can be fed back to the IAM using an iterative bi-directional soft-linking approach, which allows for model-informed improvements of the power system representation in the IAM.

This work uses pyam to implement the soft-linking method in a framework-agnostic manner. Results from any IAM can be used as starting point, as long as they are in a format compatible with pyam; and with adequate pyam-to-native-format interfaces, any power system model can be used for the highly-resolved validation and analysis. This work also uses several pyam features for data input/output, processing and visualization to streamline the implementation of the soft-linkage method.

## Outlook

### Facilitating assessments in AR6

As part of the upcoming IPCC Sixth Assessment Report (AR6), pyam has facilitated increased coordination and consistency across the analysis and data processing steps. In addition to being utilized by authors to generate key figures in the report ( link to the repository), pyam is a critical component to the overall climate assessment pipeline utilized by AR6 authors across Working Groups (I & III). All scenario data is supplied in accordance with the IAMC data format to ensure interoperability. The emissions data is then read in using pyam. Emissions data is processed using the open-source software packages aneris ^32^ and silicone ^33^ before it is run using probabilistic reduced-complexity climate models managed through the package OpenSCM-Runner ^34^.

All of these programs natively use the IAMC timeseries data format and pyam serves as the programmatic interface between the integrated-assessment scenarios and the climate model processing. The pyam validation features allows for easily checking that the minimum set of emissions data exists for each scenario, ensuring that no essential data is missing. While pyam is used for much of the pre- and post-processing, some analysis steps directly use pandas or scmdata ^22^ because they are better suited to processing large volumes of probabilistic climate data. The scripts will be released upon publication of the AR6.

### Connection to other data resources

As a next step for increasing the usefulness of the pyam package, we intend to implement additional connections to data resources: First, discussions have started with the maintainers of the Open Energy Platform (OEP) to develop an interface to their database infrastructure and related tools. Second, the just-starting Horizon 2020 project *European Climate and Energy Modelling Forum* (ECEMF) will also rely on the pyam package and the underlying data model to implement linkages between modelling frameworks and make scenario results available to stakeholders and other researchers.

### Community growth and package development

To make the development of an open-source, collaborative package like pyam sustainable over an extended period of time, it is vital to have several developers and core contributors to implement feature proposals, review pull requests and respond to bug reports. At the same time, there is an important role for (non-expert) users: suggesting new features to improve the usefulness of the package, contributing to the development of tutorials, and answering questions from new users via the community Slack channel and mailing list.

The just-starting Horizon 2020 project *European Climate and Energy Modelling Forum* ( ECEMF) will develop model linkages and tools based on or compatible with the pyam package. By virtue of being applied in several ongoing Horizon 2020 projects and the IPCC AR6 process, we are confident that the package will attract new users and continuously evolve to meet changing requirements for scenario analysis and data visualization. At the same time, the solid foundation of continuous-integration workflows, comprehensive test coverage and detailed documentation minimize the risk of inadvertently breaking existing scripts and causing frustration amongst the existing user base.

### Facilitating best practices of scientific software development and open science

This manuscript introduces the pyam package, a Python toolbox bridging the gap between scenario processing solutions that are fully customized to specific integrated assessment or macro-energy modelling frameworks, on the one hand, and general-purpose data processing and visualization packages, on the other hand.

We believe that this package can enable the adoption of best practices for scientific software development and facilitate reproducible and open science through several avenues: First, an intuitive interface and the many tutorials make it easy for non-expert users to switch from analysis using Excel spreadsheets to scripted workflows. Second, by removing clutter from scripts thanks to a well-structured and stable API, pyam allows to write more concise workflows. Thereby, scenario processing will become easier to understand, which can increase the transparency and reproducibility of the scientific analysis. Third, by implementing a generic and widely adopted data model with interfaces to several data resources and supporting multiple file types, the package can increase interoperability between modelling frameworks and streamline comparison of scenario results across projects and research domains.

Last, but not least: by providing a suite of domain-relevant methods based on a generic and versatile data model, it is our hope that using pyam will free up time for researchers and modellers to perform more scenario validation and analysis. This can improve the quality and relevance of scientific insights related to climate change mitigation pathways and the sustainable development goals.

## Software availability

Source code available from: https://github.com/IAMconsortium/pyam

Archived source code at time of publication: https://doi.org/10.5281/zenodo.1470400

JEL Codes: Q*, C65, C88

MSC Codes: 91B76, 68N01

**Table:** 

Package metadata
Current stable release: v1.0
License: Apache-2.0
Software code language: Python ≥ 3.7
Operating environments: Windows, Linux, Mac OS
Code versioning system: git (GitHub)
Documentation: https://pyam-iamc.readthedocs.io
Mailing list & forum: https://pyam.groups.io

## Data availability

### Underlying data

No data are associated with this article.

### Extended data

Software references: List of references for all packages and tools listed in Figure 1.

**Table:** 

Package/tool	URL
pandas	https://pandas.pydata.org/
numpy	https://numpy.org/
tidyverse	https://www.tidyverse.org/
Queryverse.jl	https://www.queryverse.org/
matplotlib	https://matplotlib.org/ ^4^
seaborn	https://seaborn.pydata.org ^5^
shiny	https://shiny.rstudio.com/
madrat	https://github.com/pik-piam/madrat ^6^
iamc	https://github.com/IAMconsortium/iamc
genno	https://genno.readthedocs.io/
mipplot	https://github.com/UTokyo-mip/mipplot ^7^
PUDL	https://catalyst.coop/pudl
PowerGenome	https://github.com/PowerGenome/PowerGenome
friendly_data	https://sentinel-energy.github.io
PowerSystems.jl	https://github.com/NREL-SIIP/PowerSystems.jl
Open Energy Platform	https://openenergy-platform.org
Spine Toolbox	https://spine-toolbox.readthedocs.io/
TIMES-VEDA	https://veda-documentation.readthedocs.io/
OSeMOSYS & otoole	http://www.osemosys.org/; https://otoole.readthedocs.io
MESSAGEix	https://docs.messageix.org
REMIND	https://www.pik-potsdam.de/en/institute/departments/ transformation-pathways/models/remind
GCAM	http://www.globalchange.umd.edu/gcam/
Mimi	https://www.mimiframework.org
GenX	https://genxproject.github.io/GenX/dev/
TEMOA	https://github.com/TemoaProject/temoa ^30^
pypsa	https://pypsa.org ^11^
PLEXOS	https://energyexemplar.com/solutions/plexos
