# Supplementary material for: pyam: Analysis and visualisation of integrated assessment and macro-energy scenarios
Source: Open Res Eur. 2021 Sep 1;1:74. Originally published 2021 Jun 28. [Version 2] doi: 10.12688/openreseurope.13633.2 (PMC10446008; doi:10.12688/openreseurope.13633.2)
Supplement: Peer review history

# Peer review: Reviewer response for version 2

**DOI:** 10.21956/openreseurope.15152.r27511

**Published:** 2021-10-06

**Authors:** Felipe Andres Feijoo Palacios

**Affiliations:** 1 Faculty of Mechanical Engineering and Naval Architecture, University of Zagreb, Zagreb, Croatia; 2 School of Industrial Engineering, Pontificia Universidad Católica de Valparaíso, Valparaiso, Chile

I want to thank the authors for addressing all my comments in an appropriate manner. I agree with the changes and responses that were provided.

Is the rationale for developing the new software tool clearly explained?

Yes

Is the description of the software tool technically sound?

Partly

Are sufficient details of the code, methods and analysis (if applicable) provided to allow replication of the software development and its use by others?

Partly

Is sufficient information provided to allow interpretation of the expected output datasets and any results generated using the tool?

Yes

Reviewer Expertise:

Energy systems, energy economic, IAM

I confirm that I have read this submission and believe that I have an appropriate level of expertise to confirm that it is of an acceptable scientific standard.

# Peer review: Reviewer response for version 2

**DOI:** 10.21956/openreseurope.15152.r27510

**Published:** 2021-09-13

**Authors:** Gauthier Limpens

**Affiliations:** 1 Institute of Mechanics, Materials and Civil Engineering (iMMC), UC Louvain, Louvain-la-Neuve, Belgium

Dear Authors,

 The comments I have addressed have been carefully taken into account and answered. I do approve the proposed version.

 Just a last minor comment: I saw some repetition (typo mistakes?), such as:

 ""to the to the" (p5)

 "describes describes" (p5)

 Thank you for your valuable work and this contribution for the scientific community.

Is the rationale for developing the new software tool clearly explained?

Partly

Is the description of the software tool technically sound?

Partly

Are sufficient details of the code, methods and analysis (if applicable) provided to allow replication of the software development and its use by others?

Yes

Is sufficient information provided to allow interpretation of the expected output datasets and any results generated using the tool?

Partly

Reviewer Expertise:

Energy system modelling

I confirm that I have read this submission and believe that I have an appropriate level of expertise to confirm that it is of an acceptable scientific standard.

# Peer review: Reviewer response for version 2

**DOI:** 10.21956/openreseurope.15152.r27509

**Published:** 2021-09-08

**Authors:** Matteo De Felice

**Affiliations:** 1 Joint Research Centre (JRC), European Commission, Petten, The Netherlands

The authors addressed all my previous comments and I think that the paper is more readable and complete in this form. I have no further comments.

Is the rationale for developing the new software tool clearly explained?

Partly

Is the description of the software tool technically sound?

Yes

Are sufficient details of the code, methods and analysis (if applicable) provided to allow replication of the software development and its use by others?

Yes

Is sufficient information provided to allow interpretation of the expected output datasets and any results generated using the tool?

Yes

Reviewer Expertise:

energy modelling, energy & meteorology

I confirm that I have read this submission and believe that I have an appropriate level of expertise to confirm that it is of an acceptable scientific standard.

# Peer review: Reviewer response for version 1

**DOI:** 10.21956/openreseurope.14704.r27184

**Published:** 2021-07-23

**Authors:** Felipe Andres Feijoo Palacios

**Affiliations:** 1 Faculty of Mechanical Engineering and Naval Architecture, University of Zagreb, Zagreb, Croatia; 2 School of Industrial Engineering, Pontificia Universidad Católica de Valparaíso, Valparaiso, Chile

The manuscript introduces a new open-source python package for data management and visualization of energy and climate related research, mainly for the IA models and community. I find it provides important value to the community. Authors do motivate the need for such a new package and the advantages of pyam over the existing packages. However, I think that in general authors can do a better job in describing the unique characteristics of Pyam. For instance, authors refer readers to the website to see a set of different plots that can be done. Yet, it would be good that such examples are described and exemplified in the manuscript. Currently, there is a single Figure (with low quality, which needs to be improved), and does not allow to realize the range of options and capabilities that pyam provides.

 Other minor and technical comments

- Authors mention that pyam allows for model-scenario comparison. How does pyam handles cases where different time scales want to be compared?
- The manuscript refers to other libraries, such as pandas or numpy. However, there are no comments regarding the version of such libraries or version of python. Are there limitations here or when installing pyam, the correct version of other libraries are also installed?  
- I am not sure that the shiny package, which is more for interactive plots and dashboards, goes along with ggplot, matplotlib, and seaborn (see Figure 1).
- The manuscript refers to direct interfaces that are provided by pyam (for instance, page 4 first paragraph after the bullet point #4). What are these interfaces? A clarification, and if possible, an example, would be appreciate it.  In the same paragraph, I would suggest removing “we hope”.
-  Page 7, first paragraph: Is it the “The code base” or “The base code”?

Is the rationale for developing the new software tool clearly explained?

Yes

Is the description of the software tool technically sound?

Partly

Are sufficient details of the code, methods and analysis (if applicable) provided to allow replication of the software development and its use by others?

Partly

Is sufficient information provided to allow interpretation of the expected output datasets and any results generated using the tool?

Yes

Reviewer Expertise:

Energy systems, energy economic, IAM

I confirm that I have read this submission and believe that I have an appropriate level of expertise to confirm that it is of an acceptable scientific standard, however I have significant reservations, as outlined above.

# Response

**Published:** 2021-08-12

**Authors:** Daniel Huppmann

**Thank you very much for this constructive review and suggestions to improve the manuscript.**

 Authors refer readers to the website to see a set of different plots that can be done. Yet, it would be good that such examples are described and exemplified in the manuscript. Currently, there is a single Figure (with low quality, which needs to be improved), and does not allow to realize the range of options and capabilities that pyam provides.

**We will make sure that the quality of the figure is improved during the revisions, and we extended the respective section of the manuscript to include the plot types. At the same time, we do believe that refering interested readers to gallery hosted on ReadTheDocs is a better approach rather than describing plots with text or showing a screenshot of the gallery page in the manuscript.**

 Authors mention that pyam allows for model-scenario comparison. How does pyam handles cases where different time scales want to be compared?

**To answer with an example: if model A has a five-year time resolution and model B only has decadal values, then the missing values will be shown as "NaN" where necessary, e.g., when displaying the timeseries data in wide format. This approach requires no assumptions and shows users where data does or not exist. Methods using the time resolution are implemented in a way that they are "smart" about missing data: for example, using the `interpolate()` function to get compute values for the year 2023 will automatically use the periods 2020 and 2025 for model A, but 2020 and 2030 for model B, even if they are compiled within one IamDataFrame object.**

 The manuscript refers to other libraries, such as pandas or numpy. However, there are no comments regarding the version of such libraries or version of python. Are there limitations here or when installing pyam, the correct version of other libraries are also installed?

**As is common practice in Python packages, the requirements of other packages are listed in the installation instruction and the file `setup.py`. We use GitHub Actions, including a nightly test, to ensure that all features work with the "oldest" explicitly supported versions as well as any new releases of the dependencies.**

 I am not sure that the shiny package, which is more for interactive plots and dashboards, goes along with ggplot, matplotlib, and seaborn (see Figure 1).

**Our aim here is to provide a broad overview of tools that are used by researchers, modellers and analyists for analysis and data visualization, so we prefer to keep it.**

 The manuscript refers to direct interfaces that are provided by pyam (for instance, page 4 first paragraph after the bullet point #4). What are these interfaces? A clarification, and if possible, an example, would be appreciate it.

**Due to the reworking of Figure 1 and the corresponding text, this part in chapter 1 was removed. The section "integration with data resources" in the chapter on the pyam package has a list of the currently implemented interfaces.**

 In the same paragraph, I would suggest removing “we hope”.

**This phrase has been removed as part of the rewriting of this section.**

 Page 7, first paragraph: Is it the “The code base” or “The base code”?

**We indeed mean the "code base", i.e., the collection of source code of the package.**

# Peer review: Reviewer response for version 1

**DOI:** 10.21956/openreseurope.14704.r27185

**Published:** 2021-07-19

**Authors:** Matteo De Felice

**Affiliations:** 1 Joint Research Centre (JRC), European Commission, Petten, The Netherlands

DISCLAIMER: I have tried to be as clear as possible, but without section and line numbers specifying the single parts of this paper was not easy. 

 This paper tries to describe a package which is definitely very powerful when analysing and visualising energy scenario data. I have used the verb 'try' because I think that the paper is underachieving in this sense: it explains in general terms the package without spending enough time on workflows and the possible use cases. Also, the paper is not easy to navigate giving the lack of section numbers. 

 Here my main comments: 

1. Who is the user of the package? It should be briefly stated in the introduction. Energy modellers? Analysists? Climate scientists who want to work on the energy domain?
2. Saying (section "Design principles, implementation and user groups") that this package could help two distinct users discriminating them only by their Python proficiency (experienced Python users or with a limited knowledge) seems rather limited. Is this package for energy modellers running their own models? Is this useful for people analysing runs launched by others? Is this for energy expert, climate experts or both?
3. When you say 'domain expertise', which domain you refer exactly?
4. In the section explaining the "pyam data model" you don't say that the package follows the IAMC data model and there is no reference to the previous section explaining it. In my opinion, in this section you should explain more clearly (as you do in the official docs, https://pyam-iamc.readthedocs.io/en/stable/data.html) how the data is structured but if you don't want to repeat the same information, you should at least inviting the reader to check the previous section ("Data models and formats used by the energy & climate modelling communities").
5. In table 1 you show an example of annual time-series data, can pyam also deal with sub-annual data? In that case, you should also show an example for that to help the reader to understand the data format.
6. This paper is not a tutorial, however it might explain better the most common workflows to really highlight how powerful it is: which steps are needed to (for example) calculate a multi-model average? Or how can users "import" its own data in the pyam format (this is not mentioned in the section "Operation and features")? How can they validate the data structures? I wouldn't include python code but at least I would explain the main steps. I know that you refer to the Jupyter notebooks in the ref. 27 but I think that the paper should help potential new users to realise the effectiveness of the package.
7. Please add a ref for European Climate and Energy Modelling Forum (e.g. grant number or official website).

Is the rationale for developing the new software tool clearly explained?

Partly

Is the description of the software tool technically sound?

Yes

Are sufficient details of the code, methods and analysis (if applicable) provided to allow replication of the software development and its use by others?

Yes

Is sufficient information provided to allow interpretation of the expected output datasets and any results generated using the tool?

Yes

Reviewer Expertise:

energy modelling, energy & meteorology

I confirm that I have read this submission and believe that I have an appropriate level of expertise to confirm that it is of an acceptable scientific standard, however I have significant reservations, as outlined above.

## References

[1] : pyam Data Model. Reference source

# Response

**Published:** 2021-08-12

**Authors:** Daniel Huppmann

**Thank you very much for this constructive review and suggestions to improve the manuscript.**

 1. Who is the user of the package? It should be briefly stated in the introduction. Energy modellers? Analysists? Climate scientists who want to work on the energy domain?

**We added a paragraph in the abstract and extended the relevant part in sections 1 and 3 to highlight that the package may be equally useful to all of these groups.**

 2. Saying (section "Design principles, implementation and user groups") that this package could help two distinct users discriminating them only by their Python proficiency (experienced Python users or with a limited knowledge) seems rather limited. Is this package for energy modellers running their own models? Is this useful for people analysing runs launched by others? Is this for energy expert, climate experts or both?

**We rephrased the introduction and this section to clarify that the package can be used at any combination of Python expertise and domain knowledge.**

 3. When you say 'domain expertise', which domain you refer exactly?

**The package can be useful for analysis in any of the domains mentioned throughout the manuscript: energy, integrated assessment, and climate modelling.**

 4. In the section explaining the "pyam data model" you don't say that the package follows the IAMC data model and there is no reference to the previous section explaining it. In my opinion, in this section you should explain more clearly (as you do in the official docs, https://pyam-iamc.readthedocs.io/en/stable/data.html) how the data is structured but if you don't want to repeat the same information, you should at least inviting the reader to check the previous section ("Data models and formats used by the energy & climate modelling communities").

**We extended this section as suggested and added cross-references to the "Data models" section.**

 5. In table 1 you show an example of annual time-series data, can pyam also deal with sub-annual data? In that case, you should also show an example for that to help the reader to understand the data format.

**We added a sentence in that "pyam data model" section to have a stronger emphasis that pyam can work with sub-annual time resolution, such as hourly data.**

 6. This paper is not a tutorial, however it might explain better the most common workflows to really highlight how powerful it is: which steps are needed to (for example) calculate a multi-model average? Or how can users "import" its own data in the pyam format (this is not mentioned in the section "Operation and features")? How can they validate the data structures? I wouldn't include python code but at least I would explain the main steps. I know that you refer to the Jupyter notebooks in the ref. 27 but I think that the paper should help potential new users to realise the effectiveness of the package.

**We added a new subsection "Getting started" to the "Operations and features" section, replacing and extending the previous section "Supported file formats and data types". This should make it easier for readers to find the answers to the questions that you raise.**

 7. Please add a ref for European Climate and Energy Modelling Forum (e.g. grant number or official website).

**Since submission of the intial manuscript, pyam has indeed assumed a larger role in the ECEMF project. We added a reference and link to this project in the Outlook.**

# Peer review: Reviewer response for version 1

**DOI:** 10.21956/openreseurope.14704.r27187

**Published:** 2021-07-09

**Authors:** Gauthier Limpens

**Affiliations:** 1 Institute of Mechanics, Materials and Civil Engineering (iMMC), UC Louvain, Louvain-la-Neuve, Belgium

The paper identifies a major problem in the energy and climate systems modelling community: the abundance of different methods for processing data. The ambition of the paper is high and the proposed pyam package aims to provide a toolbox for data processing before and after the model. Before the model, the package allows to read, structure, reference, display and check the data. After the model, the package allows to visualise and save in different formats some of the results data.

 The article is structured in 5 sections:

1. The introduction describes the trends in the literature and the gap that the pyam package seeks to fill.
2. A literature review focuses on data formats in the energy and climate systems modelling communities.
3. A presentation describing the main implementations of the model, the supported data and the usable features.
4. Examples of uses of the package in different projects and for different models
5. A conclusion in the form of an outlook of the work and the desire to continue the package development.

As pointed out in the article, most models have their own data processing routines involving a risk of error.

 The proposed contribution is justified and very ambitious. I support & congratulate the initiative. Nevertheless, the article can be improved in order to facilitate access for future users. Being outside the project, I can't find out if the package can be used for the model I am developing. However, the aim of this article is to answer this question and to motivate new users to join the community. Thus, the current version of the article doesn't fulfil its objective.

 These remarks lead me to 3 major comments:

**1. Lack of structure & consistency amongst the different sections.**

 Presenting a paper on a package or a model is very complicated as it is abstract for the reader. The objective can either be to interest new users or to be the reference for developers. My feeling is that authors mix the two with sometimes too much detail and often not enough global picture. The documentation (https://pyam-iamc.readthedocs.io/en/stable/) should meet the second expectation, and the article the first. Three ways can be used to improve the article.

 Firstly, some paragraphs to give an overview before diving into the details are missing. These paragraphs will help to understand:

- why there are 4 categories of packages (beginning of page 4)?
- why we are interested in these specific formats (end of page 4) and not others? Are these formats exhaustive and do they cover all formats? What is included in the term 'data model', just time series or also characteristics of technologies or resources?
- what are the functionalities of the pyam data model (page 7)?
- what kind of application cases has the model been used for (beginning of page 10)?

Secondly, figures would facilitate understanding with effective redundancy of information. Figure 1 is an example for the different categories.

 Thirdly, examples would facilitate understanding by illustrating the work. For example, on page 7 2nd §, an example would facilitate the understanding of the 2 different 'scenarios', or in the 'Operation and features' section, it would be interesting to apply the 3 steps to the same example to demonstrate.

**2. Quality of Figure 1.**

 The figure supports too many different messages with inconsistency of shapes used (i.e. same shape for different messages) or color. For example, bubbles can represent a package (e.g. mipplot) or a set (e.g. General-purpopse plotting packages). Another example, same color is used for fonts but also for backgrounds. What does this difference imply? What is the link between the 4 text blocks (with text blocks, I refer to blocks with text written, such as 'Visualization package ...')? Does the positioning between these 4 areas make sense? Are 'iamc' and 'genno' equally associated with the 'Data processing pakcages' block?

 This image is essential to understand the diversity of tools and the contribution of pyam. However, the image is too complex. A simplified version for a novice user is necessary to ensure the understanding of the article.

**3. Avoid constructions with superlatives without argument.**

 For example "simple and intuitive interface" should be justified. A screenshot of the interface could corroborate the term. Moreover, a scientific publication should remain an objective description of the work. The sentence "Visit the gallery and read the comprehensive plotting documentation for an up-to-date overview" sounds more like an advertisement for the work and could be revised to fit the expected structure.

 In addition, here are some minor comments:

1. Page 3 paragraph 2: 'users often write their...'. I emphasise that pre- and post-processing is a common source of error when applying a model to a real case. So another strength of your work is the user-related reduction.
2. Page 3, just after "An overview of existing packages and tools". Two examples of tool applications are listed, however, in the figure I see 4 'text blocks', I don't understand the link between the text and the figure. I recommend splitting the message in two. First, define the criteria used to categorise the models and then show several examples based on these criteria.
3. Page 4, top. It is not clear how and why the 4 categories were chosen. Are they completely exhaustive? It would be easier to use an example to show the difference between these 4 categories. Would it be possible to define the 4 categories on a flow chart showing when they are used (before and/or after the model), what they require and what their results are?
4. Page 4, next paragraph "A python package ...". The topic does not provide much information, I suggest to rephrase it (e.g. In a previous publication, the authors describes ...) and put reference [8] at the end.
5. Page 4, after "Data models and formats used...". This comment is linked to the main comment: what is meant by "data models" and "formats"? A brief definition is given and then several examples are described. However, the reader is still left wondering why these examples were chosen. Do they represent the different types of data models? I suggest that the first paragraph be expanded to better introduce the concepts of 'data models' and 'formats'.
6. Page 6, before the section "The pyam package". A concluding paragraph would make it easier to link the two sections. What should be concluded from the previous section? How does this relate to the next section? I would expect some limitations to be raised and answered in the next section.
7. Page 7, 2nd paragraph. As mentioned earlier, an example could support the two different types of "scenarios".
8. Page 7, 3rd paragraph: A general overview of the pyam data model would be useful for the reader to facilitate understanding. The figure could show, as an example, the link between structure and functionality?
9. Page 11, next paragraph 'Community growth and package development'. I recommend including the link to the slack channel and mailing list here again.

I hope that these comments could help you to improve your valuable work and advertise the open-source work you have achieved. I am convinced that the pyam package is a major scientific contribution and I hope that, after peer-reviewing, the article will reflect its quality.

Is the rationale for developing the new software tool clearly explained?

Partly

Is the description of the software tool technically sound?

Partly

Are sufficient details of the code, methods and analysis (if applicable) provided to allow replication of the software development and its use by others?

Yes

Is sufficient information provided to allow interpretation of the expected output datasets and any results generated using the tool?

Partly

Reviewer Expertise:

Energy system modelling

I confirm that I have read this submission and believe that I have an appropriate level of expertise to confirm that it is of an acceptable scientific standard, however I have significant reservations, as outlined above.

# Response

**Published:** 2021-08-12

**Authors:** Daniel Huppmann

**Thank you very much for this constructive review and suggestions to improve the manuscript. We agree that the manuscript should rather provide an overview for potential new users rather than give a comprehensive reference. Following your suggestion, we added a paragraph in the abstract and in section 1 to make it easier for readers to quickly understand if that package might be relevant for them. This should also answer the valid question by a reader like this reviewer whether pyam can be applicable for her or his modeling work.**

**Responses to individual comments:**

 1. Lack of structure & consistency amongst the different sections. Firstly, some paragraphs to give an overview before diving into the details are missing. These paragraphs will help to understand: why there are 4 categories of packages (beginning of page 4)?

**We re-worked the figure and changed this section to clarify that this categorization reflects our understanding of typical tasks and workflows when working with scenario data.**

 Why we are interested in these specific formats (end of page 4) and not others? Are these formats exhaustive and do they cover all formats?

**These are the data formats that we regularly encounter in our work. We expanded the introductory paragraph to clarify the scope.**

 What is included in the term 'data model', just time series or also characteristics of technologies or resources?

**We expanded the introductory paragraph of this section to clarify that the idea of a "data model" applies to any kind of structured, numerical data.**

 What are the functionalities of the pyam data model (page 7)?

**We added a reference to the section on data models and clarified the relation.**

 What kind of application cases has the model been used for (beginning of page 10)?

**The models used in the openENTRANCE project are used for analysing transition pathways of the European Union.**

 Secondly, figures would facilitate understanding with effective redundancy of information.

**Figure 1 is an example for the different categories. We hope that the revised Figure 1 makes it easier to grasp the various aspects of data processing, modelling and analysis.**

 Thirdly, examples would facilitate understanding by illustrating the work. For example, on page 7 2nd , an example would facilitate the understanding of the 2 different 'scenarios', or in the 'Operation and features' section, it would be interesting to apply the 3 steps to the same example to demonstrate.

**We added a section on how to get started in this section, and this includes references to several tutorials that should help a potential user to identify relevant workflows.**

 2. Quality of Figure 1. The figure supports too many different messages with inconsistency of shapes used (i.e. same shape for different messages) or color. For example, bubbles can represent a package (e.g. mipplot) or a set (e.g. General-purpopse plotting packages). Another example, same color is used for fonts but also for backgrounds. What does this difference imply? What is the link between the 4 text blocks (with text blocks, I refer to blocks with text written, such as 'Visualization package ...')? Does the positioning between these 4 areas make sense? Are 'iamc' and 'genno' equally associated with the 'Data processing pakcages' block? > This image is essential to understand the diversity of tools and the contribution of pyam. However, the image is too complex. A simplified version for a novice user is necessary to ensure the understanding of the article.

**Figure 1 has been extensively reworked based on your comments and suggestions.**

 3. Avoid constructions with superlatives without argument. For example "simple and intuitive interface" should be justified. A screenshot of the interface could corroborate the term. Moreover, a scientific publication should remain an objective description of the work. The sentence "Visit the gallery and read the comprehensive plotting documentation for an up-to-date overview" sounds more like an advertisement for the work and could be revised to fit the expected structure.

**We have revised the respective paragraphs to use a more neutral language.**

 In addition, here are some minor comments:

 Page 3 paragraph 2: 'users often write their...'. I emphasise that pre- and post-processing is a common source of error when applying a model to a real case. So another strength of your work is the user-related reduction.

**We added a following sentence in that subsection to highlight your point.**

 Page 3, just after "An overview of existing packages and tools". Two examples of tool applications are listed, however, in the figure I see 4 'text blocks', I don't understand the link between the text and the figure. I recommend splitting the message in two. First, define the criteria used to categorise the models and then show several examples based on these criteria.

**This section has been rewritten to better match the new Figure.**

 Page 4, top. It is not clear how and why the 4 categories were chosen. Are they completely exhaustive? It would be easier to use an example to show the difference between these 4 categories. Would it be possible to define the 4 categories on a flow chart showing when they are used (before and/or after the model), what they require and what their results are?

**Again, this section has been rewritten based on your comments. We have considered the idea of a flow chart but have not found a way to implement it that would (in our opinion) improve understanding without hampering other ideas that this figure should convey.**

 Page 4, next paragraph "A python package ...". The topic does not provide much information, I suggest to rephrase it (e.g. In a previous publication, the authors describes ...) and put reference [8] at the end.

**We rephrased that sentence as requested.**

 Page 4, after "Data models and formats used...". This comment is linked to the main comment: what is meant by "data models" and "formats"? A brief definition is given and then several examples are described. However, the reader is still left wondering why these examples were chosen. Do they represent the different types of data models? I suggest that the first paragraph be expanded to better introduce the concepts of 'data models' and 'formats'.

**The start of the section has been extended, and we added a new paragraph at the end of the section to better link it to the following section. The data models and formats listed here are those that we encounter in our work in our various research domains.**

 Page 6, before the section "The pyam package". A concluding paragraph would make it easier to link the two sections. What should be concluded from the previous section? How does this relate to the next section? I would expect some limitations to be raised and answered in the next section.

**We added a linking paragraph at the end of the section and extended the "pyam data model" subsection for clarity.**

 Page 7, 2nd paragraph. As mentioned earlier, an example could support the two different types of "scenarios".

**We added an example based on the CD-LINKS project.**

 Page 7, 3rd paragraph: A general overview of the pyam data model would be useful for the reader to facilitate understanding. The figure could show, as an example, the link between structure and functionality?

**We added a reference to the section on data models and clarified the relation.**

 Page 11, next paragraph 'Community growth and package development'. I recommend including the link to the slack channel and mailing list here again.

**We added the links to that paragraph.**
